# Adherence to 14-day radical cure for *Plasmodium vivax* malaria in Papua, Indonesia: a mixed-methods study

**DOI:** 10.1186/s12936-023-04578-3

**Published:** 2023-05-20

**Authors:** Annisa Rahmalia, Jeanne Rini Poespoprodjo, Chandra U. R. Landuwulang, Maya Ronse, Enny Kenangalem, Faustina H. Burdam, Kamala Thriemer, Angela Devine, Ric N. Price, Koen Peeters Grietens, Benedikt Ley, Charlotte Gryseels

**Affiliations:** 1 Timika Malaria Research Programme, Papuan Health and Community Development Foundation, Timika, Indonesia; 2 Global and Tropical Health Division, Menzies School of Health Research and Charles Darwin University, Darwin, Australia; 3 Institute of Tropical Medicine, Antwerp, Belgium; 4 Mimika District Hospital, Timika, Indonesia; 5 Paediatric Research Office, Department of Child Health, Faculty of Medicine, Public Health and Nursing, Universitas Gadjah Mada/Dr. Sardjito Hospital, Yogyakarta, Indonesia; 6 Mimika Regency Health Authority, Timika, Papua, Indonesia; 7 Centre for Epidemiology and Biostatistics, Melbourne School of Population and Global Health, University of Melbourne, Melbourne, Australia; 8 Nuffield Department of Clinical Medicine, Centre for Tropical Medicine and Global Health, University of Oxford, Oxford, UK; 9 Mahidol-Oxford Tropical Medicine Research Unit (MORU), Faculty of Tropical Medicine, Mahidol University, Bangkok, Thailand; 10 School of Tropical Medicine and Global Health, Nagasaki University, Nagasaki, Japan

**Keywords:** Primaquine adherence, Malaria radical cure, Vivax treatment, Malaria in Papua, Malaria Indonesia

## Abstract

**Background:**

Reducing the risk of recurrent *Plasmodium vivax* malaria is critical for malaria control and elimination. Primaquine (PQ) is the only widely available drug against *P. vivax* dormant liver stages, but is recommended as a 14-day regimen, which can undermine adherence to a complete course of treatment.

**Methods:**

This is a mixed-methods study to assess socio-cultural factors influencing adherence to a 14-day PQ regimen in a 3-arm, treatment effectiveness trial in Papua, Indonesia. The qualitative strand, consisting of interviews and participant observation was triangulated with a quantitative strand in which trial participants were surveyed using a questionnaire.

**Results:**

Trial participants differentiated between two types of malaria: *tersiana* and *tropika*, equivalent to *P. vivax* and *Plasmodium falciparum* infection, respectively. The perceived severity of both types was similar with 44.0% (267/607) perceiving *tersiana* vs. 45.1% (274/607) perceiving *tropika* as more severe. There was no perceived differentiation whether malaria episodes were due to a new infection or relapse; and 71.3% (433/607) acknowledged the possibility of recurrence. Participants were familiar with malaria symptoms and delaying health facility visit by 1–2 days was perceived to increase the likelihood of a positive test. Prior to health facility visits, symptoms were treated with leftover drugs kept at home (40.4%; 245/607) or bought over the counter (17.0%; 103/607). Malaria was considered to be cured with ‘blue drugs’ (referring to dihydroartemisinin-piperaquine). Conversely, ‘brown drugs,’ referring to PQ, were not considered malaria medication and instead were perceived as supplements. Adherence to malaria treatment was 71.2% (131/184), in the supervised arm, 56.9% (91/160) in the unsupervised arm and 62.4% (164/263) in the control arm; p = 0.019. Adherence was 47.5% (47/99) among highland Papuans, 51.7% (76/147) among lowland Papuans, and 72.9% (263/361) among non-Papuans; p < 0.001.

**Conclusion:**

Adherence to malaria treatment was a socio-culturally embedded process during which patients (re-)evaluated the characteristics of the medicines in relation to the course of the illness, their past experiences with illness, and the perceived benefits of the treatment. Structural barriers that hinder the process of patient adherence are crucial to consider in the development and rollout of effective malaria treatment policies.

**Supplementary Information:**

The online version contains supplementary material available at 10.1186/s12936-023-04578-3.

## Background

Outside of sub-Saharan Africa, the burden of malaria is falling, although the relative proportion of malaria due to *Plasmodium vivax* is rising [1]. Malaria control efforts focused on reducing *P. vivax* infections and their recurrences will consequently be critical in achieving malaria elimination targets [2, 3]. *Plasmodium vivax* is particularly difficult to eliminate since it forms dormant liver stages (hypnozoites) that can reactivate weeks to months after the initial infection causing relapses and maintaining ongoing transmission. For more than half a century, primaquine (PQ) has been the only widely available drug that kills *P. vivax* hypnozoites [4, 5]. Currently PQ treatment is provided over the course of 14 days in combination with a shorter 3-day course of schizontocidal treatment to eliminate the blood stages of *P. vivax* (called radical cure) [6]. While well tolerated in most recipients, PQ can cause severe haemolysis in individuals with glucose-6-dehydrogenase (G6PD) deficiency [7]. The World Health Organization (WHO) recommends screening patients for G6PD deficiency prior to PQ based radical cure [8]. In practice, G6PD testing is rarely available resulting in under-prescription of PQ for fear of haemolysis [9, 10]. When radical cure is prescribed, the long course of PQ can undermine patient adherence impacting on drug effectiveness [11].

Studies about patient adherence to antimalarials yielded varied results related to patient characteristics, the nature of their consultation with the provider, and methodological variations of the study [12]. Assessment of adherence relied on subjective measures such as self-reporting or a combination of self-reporting and more objective measures such as pill count, presenting empty pill bags, or directly measuring the level of PQ in the blood [13–16]. Treatment supervision to improve adherence has been studied, including in the trial where this study is embedded [17, 18]. As such, trial procedures can have an impact on adherence outcomes [12].

Decision on whether to adhere to treatment is often influenced not only by individual but also societal factors [14, 19]. Community perceptions of malaria, its recurrence and treatment influence the uptake of and adherence to appropriate malaria treatment [11, 20]. In the high malaria endemic area of Mimika, Papua, Indonesia health provider factors (such as prescriber adherence to treatment protocol) were found to influence uptake of malaria testing and treatment service and treatment-seeking behaviour [21, 22]. However, little is known about the socio-cultural factors driving treatment-seeking behaviour and adherence in the complex context of Papua.

A better understanding of adherence to treatment at the community level inform context-specific approaches to health care delivery to ensure that they will have the greatest possible impact on patient outcomes and the burden of disease. This study assessed malaria perceptions, treatment-seeking behaviour, and adherence to 14-day radical cure for *P. vivax* malaria in Mimika, Papua, Indonesia.

## Methods

### Study context

**Fig. 1:**
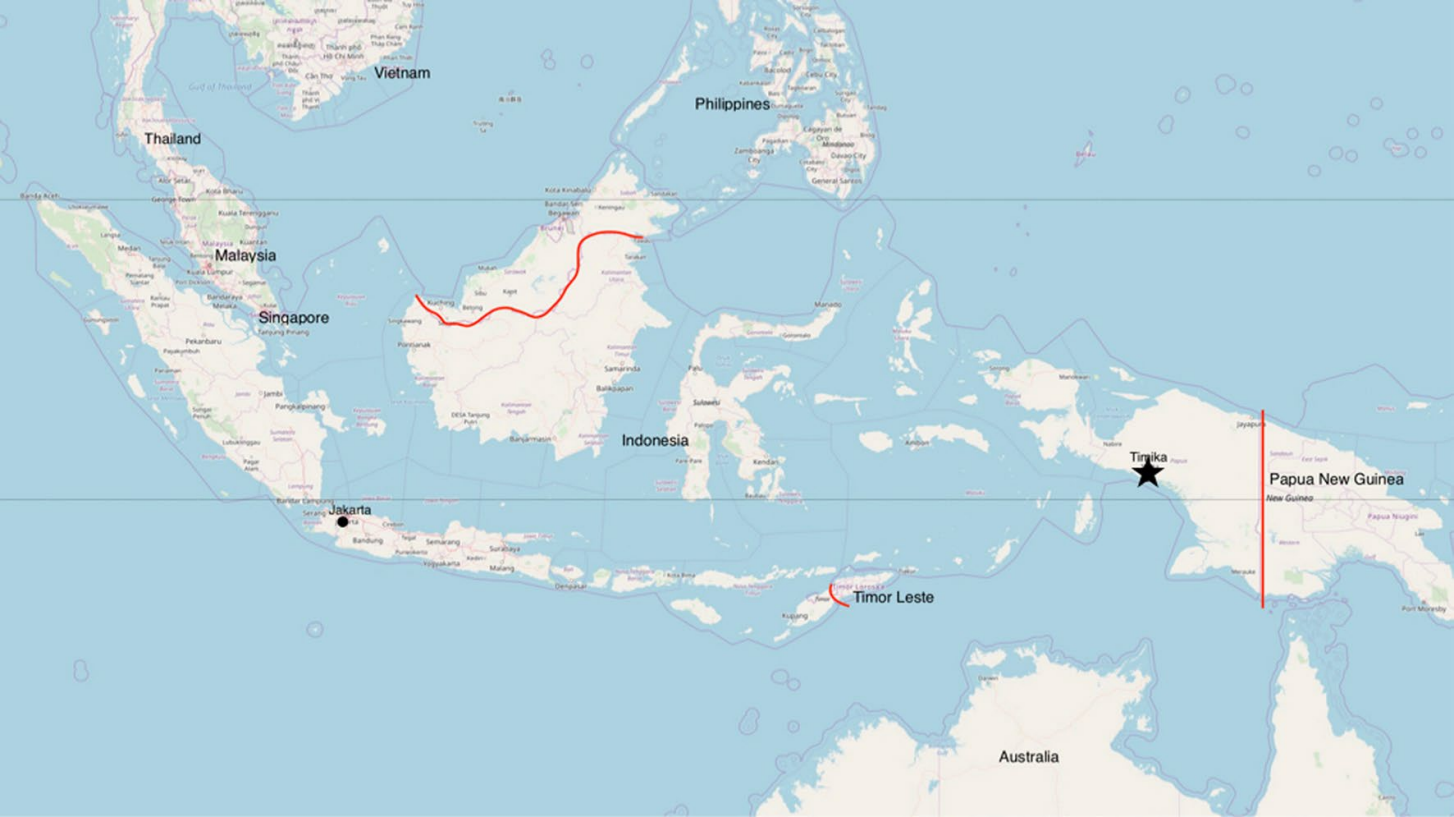
Map indicating the location of Timika (marked with a star). Map source: gpspointsplotter.com

This mixed-methods study was embedded in a cluster-randomized, controlled, open label trial in Mimika District in Papua, Indonesia (Fig. 1) [18]. Patients with microscopy confirmed malaria were enrolled into the trial at 10 local public healthcare centres. All patients, irrespective of malaria species, were treated with dihydroartemisinin-piperaquine (DP) followed by 14 days of PQ after screening for haemoglobin (Hb) and G6PD levels two days after diagnosis and initial treatment [18]. The PQ used in this study was administered at a total dose of 7 mg/kg, which, although recommended by the WHO for areas with high relapse risk, is not current policy in Indonesia, which recommends a total dose of 3.5 mg/kg (equivalent to a dose of 0.25 mg/kg per day for 14 days) for patients with *P. vivax* and *Plasmodium ovale* malaria (single or mixed infection). Patients with *Plasmodium falciparum* malaria were treated with DP and a single dose of 15 mg PQ on the day of diagnosis. In the current clinical practice outside of this trial, malaria treatment is given without testing Hb levels and G6PD activity.

The study area was divided into 21 clusters, which were randomized to a supervised and unsupervised PQ arm. In the *supervised* arm, participants were provided with two daily doses of PQ on alternate days, one dose to be taken in front of the study team and the other the next day and this was repeated for seven visits (14 PQ doses in total). In the *unsupervised* arm, participants were provided with 14 PQ doses while receiving a more thorough explanation on the importance of finishing the PQ course than is common in clinical practice. At each monthly visit, a microscopic blood film examination was undertaken to detect recurrent parasitaemia. All patients were provided with a phone number to contact the study team if they had symptoms suggestive of a malaria episode and/or a haemolytic event between the scheduled visits.

Seven months after the first enrollment in the *supervised* and *unsupervised* trial arms, additional patients from all 21 clusters were enrolled into a third, non-randomized, observational *control* arm. Participants received standard care (species-specific treatment and low dose PQ), as per usual practice without the extensive trial-specific follow up. Participants of the *control* arm were only followed up once six months after enrolment. Arm specific procedures are outlined in Table 1.

**Table 1:** Treatment and supervision procedures of study arms

	Supervised		Unsupervised	Control
Total PQ dose (radical cure)	7 mg/kg (max. 2 tablets/day) over 14 days			3.5 mg/kg (max. 1 tablet/day) over 14 days
PQ prescription	All patients irrespective of malaria species			Only patients with *P. vivax* and *P. ovale* (single or mixed) species received radical cure *P. falciparum* patients received single dose 15 mg PQ
Treatment steps and supervision	Supervised DP for 3 days followed by 14 days of daily PQ supervised on alternate days		Supervised DP for 3 days followed by unsupervised PQ	Supervised first dose of DP followed by unsupervised DP and PQ
Follow-up visits	D1, D2, D4, D6, D8, D10, D12, D14, D16, M1, M2, M3, M4, M5, M6		D1, D2, D16, M1, M2, M3, M4, M5, M6	M6

PQ, primaquine; mg, milligram; kg, kilogram; max., maximum; DP, dihydroartemisinin piperaquine; D, day; M, month

### Study design

**Fig. 2:**
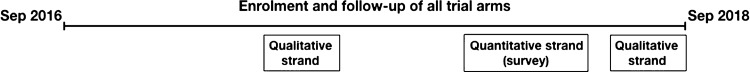
Timeline of mixed-methods study within trial period

A mixed-methods study was conducted using qualitative and quantitative research methods for data triangulation and complementarity purposes. The study had a sequential design, comprising of three consecutive strands, which in standard annotation can be presented as [qual$\to$QUAN$\to$qual] [23]. Figure 2 explained the data collection timeline within the trial.

### Quantitative strand

#### Data collection

A structured questionnaire was developed for the quantitative patient survey based on insights from the exploratory qualitative strand. The questionnaire was pre-tested in the same setting. The survey aimed to assess adherence to PQ and other malaria treatments, perceptions of malaria and its recurrences, treatment-seeking behaviour, and perceptions of the trial. Data collection was conducted in collaboration with AKVO (https://akvo.org, last accessed on 12.12.2022) using software installed on electronic tablets for automatic data entry and continuous monitoring to ensure data quality. Six research assistants with knowledge of the local culture and some of the local languages who had been involved in the clinical trial were recruited and trained as enumerators.

#### Sampling

**Fig. 3:**
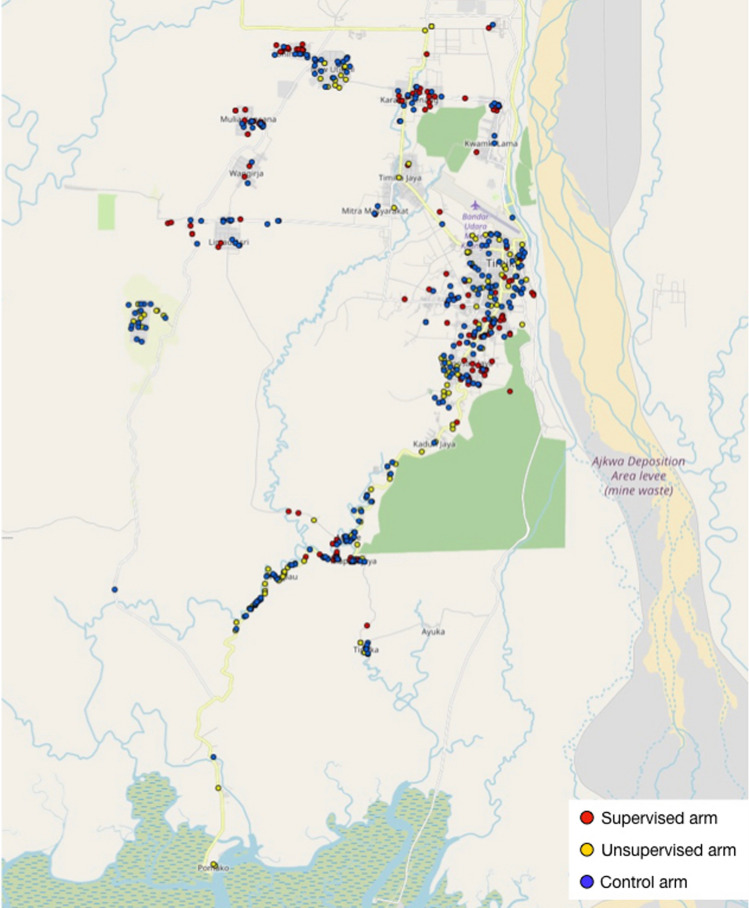
The spread of survey participants in and around the town of Timika. Map generated on gpspointplotter.com

This study was embedded in a trial conducted in 21 clusters across different districts in and around the town of Timika (Fig. 3). All patients registered in the trial database were included in the quantitative strand of this study. Enumerators first attempted to contact patients by telephone to arrange an appointment for an interview in their homes; however, interviews were also conducted at participants’ school or workplace. Patients who could not be contacted by phone or who did not have a mobile phone were visited without an appointment.

#### Data analysis

Preliminary data cleaning was conducted concurrently to the survey to resolve data entry errors and double entries using automated data entry software (Akvo™, Amsterdam, The Netherlands). Subsequent cleaning and analyses were conducted in R (R version 3.3.0, The R Foundation for Statistical Computing). Descriptive statistics were used to indicate proportions which were compared by the chi-square test. Post-hoc analyses using Marascuilo procedure were performed to test which specific proportions are different from each other in multiple comparisons of proportions.

### Qualitative strand

#### Data collection

The qualitative strand explored people’s perceptions of malaria, malaria treatment-seeking behaviour, adherence to malaria treatment, and perceptions of the ongoing trial. Trained Indonesian social scientists (AR, CL) collected data through interviewing and participant observation with community members of all ethnicities.

#### Sampling

A theoretical sampling strategy gradually included informants purposively selected in relation to emerging results based on malaria status, ethnicity, age, occupation, including trial participants and household members, other members of target communities, trial field supervision staff, healthcare staff from the various healthcare facilities in this region (not limited to participating facilities), and alternative healthcare providers (e.g., traditional health providers).

#### Data analysis

The iterative nature of qualitative data collection required data collection tools to be adapted continuously by intermittent analysis of raw data. Abductive analysis—a continuous interplay between emerging findings and theory—resulted in a theoretical model of factors influencing adherence to radical cure in this context [24]. NVivo 12 qualitative data analysis software (QSR International Pty Ltd. Cardigan, UK) was used to construct a final coding tree, which was used for thematic content analysis of all raw data and analytical memos.

### Concept operationalization

#### Ethnicity

The Island of Papua has diverse ethnicities, both internal migrant (from the western islands of Indonesia) and indigenous groups. In Indonesia, the concept of “indigenous” is highly contested because of the government view that everyone is indigenous, however, in Mimika regency, the sense of ethnic identity differentiating Papuans and non-Papuans are widely held [25, 26]. Among the Papuans, the many ethnic groups can be divided into highland and lowland Papuans based on linguistic and genetic characteristics [26–28]. In Mimika, the traditional owners of the lands that make up the regency from coastline to mountains are the Kamoro (a lowland ethnic group) and Amungme (a highland ethnic group) peoples. The non-Papuans are commonly called ‘*pendatang’* or the ‘incomers,’ regardless of how many generations they have lived in the area. They are comprised of different ethnic groups from various parts of Indonesia and arrived in Mimika regency through different modes of migration for assorted reasons for decades [26, 29]. In this study, the study population was classified into three categories: (1) highland Papuans, (2) lowland Papuans, and (3) non-Papuans as has been done in other malaria studies [18, 21, 30].

#### Adherence

In this study, adherence was defined as the aggregate of several factors quantified in the questionnaire. A patient was considered adherent if they: (1) stated to have finished all the drugs given during the trial/in the last malaria episode AND (2) mentioned ‘blue drugs’ PLUS any of the following: ‘brown drugs,’ ‘small drugs,’ ‘spleen drugs,’ or ‘primaquine’ when asked which drugs they have finished. Several variables potentially influencing self-reported adherence were considered, including trial arm, ethnicity, initial diagnosis during trial, and time between end of trial and interview.

#### Public health facility

Public health facilities were defined as primary health care clinics and the hospital, both of which were owned and run by the government. These included Puskesmas (*public health center*), Pustu (*satellite health center*), and the general hospital. For the Puskesmas and Pustu, priority access is given by area of residence. Registered Indonesian citizens (presenting a citizen card) can apply for national health insurance to get free basic healthcare at government health facilities and certain private health facilities that collaborate in the national health insurance system. In this system, clients are assigned one clinic as their primary health care provider—for most people this will be the Puskesmas nearest to their registered address.

#### Private health facility

Private health facilities were defined as non-governmental primary health care clinics and hospitals and these included malaria control clinics and the Caritas hospital (both owned by the biggest mining company in the area) as well as the private practices of local doctors and midwives. The mining company provides free access to its health facilities for Kamoro and Amungme peoples as well as five other highland ethnic groups regarded to be directly affected by mining operations (Damal, Dani, Mee, Moni, and Nduga peoples).

#### Ethical considerations

The trial and the mixed-methods study were conducted according to the principles stated in the Declaration of Helsinki (Ethical Principles for Medical Research Involving Human Subjects) as amended in 2008, all applicable regulations and according to established international scientific standards. The study protocol was reviewed and approved by the ethical committees of the Faculty of Medicine Gadjah Mada University in Indonesia (KE/FK/522/EC/2016), the Human Research Ethics Committee (HREC) of the Northern Territory (NT) Department of Health and Menzies School of Health Research in Australia (15-2517), and the Institute of Tropical Medicine in Belgium (1163/17).

For the qualitative strand, oral consent was collected from all participants and, for minors, from a legal guardian. For the quantitative survey, written informed consent was obtained from all participants or legal guardians in case they were underage. Illiterate participants willing to participate provided consent by a fingerprint in the presence of a witness.

## Results

### Study participants

#### Qualitative strand

In total 47 interviews (both in-depth and informal) and 12 observations were conducted and transcribed. Audio-recorded interviews were transcribed ad verbatim and informal conversations were summarized.

#### Quantitative strand

**Fig. 4:**
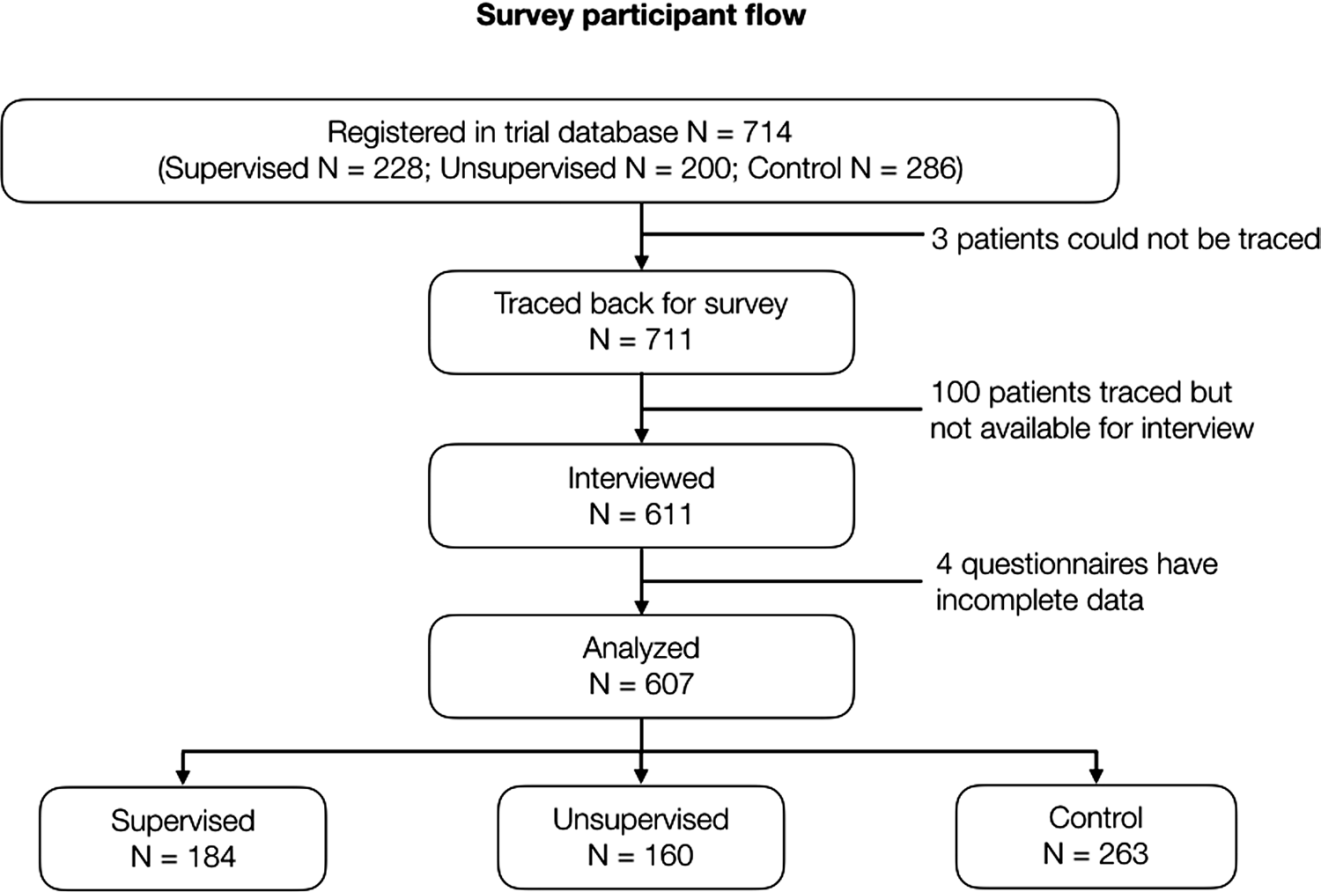
Survey participant flow for the quantitative strand

85.6% (611/714) of trial participants from all three arms participated in the survey. After data cleaning four participant records were excluded due to missing answers. In total 607 questionnaires were analysed (Fig. 4) and 50.4% (306/607) of all respondents were women. The median age was 17 years old (interquartile range (IQR): 8–33); Additional file 1: Table S1. Of all participants, 16.3% (99/607) were *highland Papuans* including 11.0% (67/607) who self-identified as Dani, 24.2% (147/607) were *lowland Papuans* with 15% (93/607 Kamoro, and 59.5% (361/607) were *non-Papuans* including 13% (77/607) Kei, from the neighbouring archipelago of Maluku, and 15% (88/607) Java/Sunda, the largest ethnic groups in Indonesia.

### Malaria types, etiologies, and recurrences

#### Malaria types

Participants had a clear understanding of the presence of various kinds of malaria in their region. The types of malaria that people referred to were derived from medical terms that healthcare workers at public health centres used amongst themselves and in communication with patients, such as malaria *tropika* and malaria *tersiana.* Healthcare workers used the term *tropika* to refer to *P. falciparum* and *tersiana* for *P. vivax* malaria. In terms of symptoms, patients perceived *malaria tersiana* to cause body pains, prolonged fevers, and the inability to do any work or other activities. Malaria *tropika* was perceived to cause headaches, a shorter febrile illness, and a lack of appetite. Some participants regarded malaria *tersiana* as being more serious due to its repeated episodes and it being perceived to make one weaker, despite having milder or no symptoms. A community member explained: *“for malaria tersiana, we don’t really get a fever, but it is very serious because it takes a long time.”* In the survey, the proportion of respondents perceiving one malaria type as more severe than the other was similar with 44.0% (267/607) perceiving *tropika* as more severe vs. 45.1% (274/607) perceiving *tersiana* as more severe. 47.9% (291/607) of respondents acknowledged the possibility of asymptomatic malaria while 28.8% (175/607) perceived it to be impossible to have malaria in the blood without symptoms (Table 2).

**Table 2:** Malaria types, etiologies, and treatment perceptions mentioned in survey (N = 607)

	Frequency (n)	Proportion (%)
*Knowledge of several types of malaria*		
Awareness of different malaria species	587	96.7
*Types of malaria mentioned*		
Malaria *tersiana*	573	94.4
Malaria *tropika*	571	94.1
Mixed infection	25	4.1
Malariae malaria	18	3.0
Bone malaria	17	2.8
Others (*quartana*, haemorrhagic fever, batana, gamete)	28	4.6
*Perceived malaria severity*		
*Tersiana* is more severe	267	44.0
*Tropika* is more severe	274	45.1
Other types of malaria are more severe	14	2.3
Do not know	18	3.0
No answer	34	5.6
*Perception of the cause of a malaria episode*		
Fatigue	284	46.8
Mosquito bite	224	36.9
Weather-related (rain/cold/heat/etc.)	138	22.7
Eating too late in the evening	107	17.6
Worked too hard	57	9.4
Food-related (ate cold food/street food/cane and coconut)	6	1.0
Not using bed net	31	5.1
*Perception of malaria illness*		
Possible to have malaria in blood and feeling healthy	291	47.9
Not possible to have malaria in blood and feeling healthy	175	28.8
Do not know	141	23.2
*Perception of malaria treatment effectiveness*		
Make body feel better	581	95.7
Does not make body feel better	21	3.5

#### Malaria aetiologies

Only 36.9% (N = 224) of respondents mentioned mosquito bites as the cause of their last malaria episode, as participants perceived malaria episodes to have multiple etiologies. A malaria episode could be caused by being near bodies of water (e.g., rivers, ditches) or triggered by working too hard and not eating well. Among survey respondents (Table 2), 46.8% (N = 284) reported that the fatigue caused by working too hard made people vulnerable to malaria, especially in combination with adverse weather conditions (22.7%, N = 138). The physical conditions that were perceived to favor a malaria episode included lower general body fitness and a weakened immune system.

#### Perception of recurrences

Participants frequently experienced repeated episodes of malaria, but there was no perceived difference between a primary malaria episode and a relapse. The length of time between one malaria episode and another was not considered an indicator of whether a malaria episode was a reinfection or a relapse. Papuan populations who had lived in the area for generations reported that repeated malaria episodes cause belly swelling due to spleen enlargement. They told their children not to hit someone in the belly to avoid breaking the spleen, and the term *“poro limpa”* (literally “spleen belly”) is used to mock people with rotund midsections.

Participants who were interviewed during the trial mentioned repeated malaria episodes were due to not finishing the drugs. As expressed by a male patient from the supervised trial arm: *“Probably the malaria (parasite) just fainted. So, if we do not finish the medicines, it would be stronger in our body.”* In the survey, most respondents agreed that recurrences are possible after a fully treated malaria infection (71.3%, 433/607) (Table 3).

**Table 3:** Self-reported recurrence of trial participants (N = 607)

	Frequency (n)	Proportion (%)
*Perception of recurrence*		
Malaria can come back after finishing medication	433	71.3
Malaria cannot come back after finishing medication	162	26.7
Do not know	12	2.0
*Self-reported recurrence during trial period (participant recall)*		
Had another malaria episode during the trial	139	22.9
Did not have another malaria episode during the trial	439	72.3
Do not remember malaria episode	2	0.3
Recurrences recorded during the trial* (N = 409)		
1 or more recurrences	174	42.5
No recurrences	235	57.5

*Recurrences during the trial follow-up period were only recorded in the supervised and unsupervised arms; in the control arm these data were not recorded as they were only visited once at the end of the 6-month period since enrolment

### Therapeutic itineraries

#### Biomedical and alternative treatment

The qualitative analyses showed that malaria was frequently self-diagnosed based on symptom recognition, with fever being the most widely reported characteristic of malaria. Associated with self-diagnosis was the common use of various home treatments (including paracetamol, traditional treatments, and leftover antimalarials) to counter the first signs of malaria. In the survey (Table 4), most participants reported that prior to visiting a health service, they relieved symptoms with leftover drugs kept at home (40.4%, 245/607) or with non-prescription drugs purchased over the counter (17.0%, 103/607). Visiting a health facility was the second stage of the therapeutic itinerary, usually occurring later than 24 h after symptom onset. In the qualitative interviews, people reported this delay to be intentional to increase the likelihood that their malaria test would be positive. The test was thought to be negative if done in the initial stages of illness onset, causing disappointment because this meant they would get no treatment to take home. Negative test results, therefore, drove people to visit different health centres to disprove the initial test results. Health care workers often have no explanation as to the cause of fever in the event of negative malaria test results.

**Table 4:** Treatment-seeking behaviour and drug of choice for malaria symptoms (N = 607)

	Frequency (n)	Proportion (%)
*Steps of treatment before visiting health provider (can give multiple answers)*		
Use remaining drugs at home	245	40.4
Do nothing	214	35.3
Buy over-the-counter drugs (pharmacy/kiosk/etc.)	103	17.0
Herbal treatments	59	9.7
Blood letting	26	4.3
Others (pray, drink, get warm, apply wet cloth, etc.)	32	5.3
*Time of visit to health provider*		
Same day as onset of symptoms	44	7.2
Day after onset of symptoms	131	21.6
Two days or more after onset of symptoms	425	70.0
Do not remember	6	1.0
*First choice of health provider*		
Public health centre	415	68.4
Malaria control clinic*	138	22.7
Private clinics/private practice	17	2.8
General hospital	6	1.0
Caritas hospital*	21	3.5
Others (over-the-counter drugs, community health worker, etc.)	8	1.3
Do not know	2	0.3
*First choice of drug (* *can give multiple answers)*		
Blue drugs	537	88.5
Brown drugs [without blue drugs]	334 [21]	55.0 [3.5]
Green drugs	64	10.5
Yellow drugs	136	22.4
Primaquine	46	7.6
Spleen drugs	7	1.2
Small drugs	24	4.0
Paracetamol	111	18.3
White drugs	149	24.5
Chloroquine/quinine	11	1.8
Antibiotics	4	0.7
Supplements/vitamins	7	1.2
Herbal e.g., papaya leaves, cat’s whiskers, …	56	9.2
*Use of herbal medication*		
Oral/topical, complementary to medical drug	46	7.6

*Funded by the mining company; free healthcare for seven ethnic groups directly affected by mining operations

#### Public and private health facilities

Significant differences between the experiences of care at the government public health centre and at the malaria control clinics were reported. According to community members and healthcare workers, tests were only conducted if the clinical symptoms warranted such a test at public health centres; while at malaria control clinics, people were tested for malaria immediately and regardless of symptoms, which increased the perceived quality of care. Participants were less certain that medication would be prescribed at the public health centre and did not trust its quality of service and drugs. Malaria control clinics were also perceived to be less crowded and have shorter waiting times and longer opening hours into the afternoon, which suited people who farm in the morning.

Despite the perception of less favourable service at government public health centres, 68.4% (415/607) stated that government public health centres were their first choice of health provider, whereas 22.7% (138/607) reported that a malaria control clinic was their first choice (Table 4).

### Perceptions of anti-malaria medicines

#### DHA-piperaquine (DP)

The standard schizontocidal treatment, DP, was locally known as the ‘blue drug’ due to its colour. The blue drug invoked confidence, which was related to expectations of the type of malaria it targeted, its perceived side effects, its perceived effectiveness, and the duration of the treatment. Some people remembered the first time in 2006 when DP became widely used and considered it to be highly efficacious in relieving symptoms compared to the previous malaria drugs. Over time, this treatment has become socially acceptable, and adhering is considered socially responsible—the mother who gives DP to her child is seen as providing ‘good’ care for her children. The treatment was reported to produce side effects such as nausea, and the large size and the number of tablets taken per day (based on bodyweight, an adult typically needs to take 3–4 tablets/day) was considered unpleasant, but these aspects had become part of the common knowledge on how DP worked and were accepted as part of the package of care that comes with the ‘blue drug’ regimen.

#### Primaquine (PQ)

Unlike the ‘blue drug,’ PQ was not identified as being a malaria drug. Among some indigenous populations, PQ is called the ‘spleen drug,’ while DP is called ‘malaria drug.’ PQ was also commonly referred to as the ‘brown drug’ and its earthy colour did not stand out as much as the bright blue DP tablets. PQ tablets are smaller than DP, a characteristic which was associated with the drug being less ‘strong’ and less ‘important.’ The prescription of PQ is inconsistent, since not all malaria patients would get it, or different durations would be prescribed. While patients were advised to finish all PQ tablets, the role of the drug for malaria was often not stated. Inconspicuous drug appearance, combined with inconsistent prescription of PQ and its long duration of treatment resulted in the meaning of PQ being re-interpreted at community level and harmonized with perceptions of vitamins or supplements. As recalled by one trial participant: *“…yes, just vitamin, the small one, for malaria parasite, the colour was brown. I just took one tablet per night.”*

These qualitative findings were confirmed in the survey in which 88.5% (537/607) of respondents provided the ‘blue drugs’ as the first answer when asked about their first choice of malaria drugs, followed by 55.0% (334/607) mentioning the ‘brown drugs’ (Table 4). Only 3.5% (21/607) referred to the ‘brown drugs’ without the ‘blue drugs.’ Some patients mentioned ‘green drugs’ (10.5%; N = 64). The first DP tablets distributed in the area in 2006 were green, so it could be assumed that green drugs refer to DP as well. Other names/colours that could indicate PQ were ‘yellow drugs,’ ‘small drugs,’ and ‘spleen drugs.’ A small proportion of participants (7.6%; N = 46) mentioned ‘primaquine’ by name.

#### Alternative therapies

Almost all participants believed in the effectiveness of biomedical malaria treatment and the use of traditional or herbal therapeutics was seen as complementary and used to relieve symptoms instead of as a cure. A variety of nettles were applied on the skin, generating a sensation of itching or heat to alleviate the feeling of joint pain and general body discomfort. Some people grew them in their garden. One type of nettle (*Laportea decumana*) was sold at the markets, typically by vendors from the highland Papuan populations. Blood letting was another customary practice among highland populations to reduce the feeling of pain in certain areas of the body (for example, blood letting from the forehead in the case of headaches).

### Malaria treatment adherence

#### Perceptions and experiences with treatment completion

In line with the perceptions of malaria medicines, completion of malaria treatment was only associated with DP, and not with PQ. Furthermore, the long duration of PQ treatment, that exceeded resolution of malaria symptoms, reinforced the belief of the potency of DP and the lack of importance of PQ. When asked which drugs participants received in the last malaria episode during the trial, 86.8% (527/607) mentioned the ‘blue drugs’ and 58.8% (357/607) the ‘brown drugs (Table 5).’ A follow-up question to the participants who claimed that they had only completed some of the drugs (N = 88) found that 86.4% (N = 76) of participants specified finishing the ‘blue drugs’ but only 22.7% (N = 20) the ‘brown drugs.’

**Table 5:** Perception and experience of completing course of drugs (N = 607)

	Frequency (n)	Proportion (%)
*Perception on completing course of drugs*		
Should finish all drugs prescribed/given	522	86.0
Should stop once you feel better	79	13.0
Depending on the type of malaria	2	0.3
Do not know	4	0.6
*Drugs received during the trial/in the last malaria episode (can have multiple answers)*		
Blue drugs	527	86.8
Brown drugs	357	58.8
Yellow drugs	136	22.4
Small drugs	32	5.3
Spleen drugs	8	13.2
Primaquine	41	6.8
Green drugs	39	6.4
Paracetamol (incl. Dumin*/drugs for fever)	212	34.9
White drugs	136	22.4
*Drugs finished during the trial/in the last malaria episode*		
All drugs ^b^	511	84.2
Some of the drugs ^a c^	88	14.9
Did not finish any drugs ^c^	6	1.0
Do not remember	2	0.3
*Which drugs were finished (asked to those who finished some of the drugs, N = 88)* ^a^		
Blue drugs	76	86.4
Brown drugs	20	22.7
Small drugs	2	2.3
Primaquine	4	4.6
Yellow drugs	5	5.7
Green drugs	3	3.4
Paracetamol (incl. Dumin*)	6	6.8
White drugs	6	6.8
Others (antibiotics/drugs for nausea)	2	2.3
*Reasons for finishing the drugs (asked to those who finished all drugs, N = 511)* ^b^		
Wanting to get better	298	58.3
Not wanting to get malaria again	126	24.7
The doctor/trial staff told me to	87	17.0
To stay healthy after feeling better	12	13.6
To kill the germs/the illness	5	1.0
Liking taking drugs	2	0.4
Considering it poor practice not to finish the drugs	1	0.2
I do not know	3	0.6
*Reasons for not finishing (asked to those who finished some of the drugs and who did not finish any drugs, N = 94)* ^c^		
Feeling better/no more fever or nausea	69	73.4
Forgetting to take the drug	14	14.9
The taste of the drug/Not liking drugs	12	12.8
Nausea/dizziness	5	5.3
Others (course was too long/took other drugs/someone told me not to/prefer the blue drugs)	5	5.3

*A local brand of paracetamol

^a^ Which drugs were finished (asked to those who finished some of the drugs, N = 88)

^b^ Reasons for finishing the drugs (asked to those who finished all drugs, N = 511)

^c^ Reasons for not finishing (asked to those who finished some of the drugs and who did not finish any drugs, N = 94)

Completing the full course of treatment was perceived as important by 86.0% (522/607) of participants and 84.2% (N = 511) stated they finished all drugs in their last malaria episode (Table 5). ‘Wanting to get better’ was the main motivation for completion (58.3%; 298/511), followed by ‘not wanting to get malaria again’ (24.7%; 126/511). The most cited reason for not completing treatment was feeling better or no longer having fever (73.4%; 69/94). In addition, perceptions of what malaria treatments were needed and were received during the trial varied, indicating that adherence to the *package* of treatments included in radical cure was not straightforward.

#### Aggregated adherence predictors

Trial participants in all three arms reported different adherence to the treatment. Measure of adherence to calculate predictors was derived from the survey questions on completion of both PQ and DP. Adherence was low in all arms: 71.2% (131/184) in the supervised arm, 56.9% (91/160) in the unsupervised arm, and 62.4% (164/263) in the control arm; overall difference p = 0.019, Table 6. Adherence did not differ between patients who were interviewed in the survey within the 6 months trial (before the last follow-up visit, or for control arm, the only follow-up visit) or after (p = 0.417, Table 6).

**Table 6:** Aggregated measure of adherence* in different study arms and ethnic groups (N = 607)

	Adherence N (%)	Non-adherence N (%)	p-value**
*Study intervention arm*			0.019
Supervised (N = 184)	131 (71.2)	53 (28.8)	
Unsupervised (N = 160)	91 (56.9)	69 (43.1)	
Control (N = 263)	164 (62.4)	99 (37.6)	
*Malaria species at trial enrolment*			0.084
*P. vivax* single/mix (N = 260)	179 (68.8)	81 (31.2)	
*P. falciparum* (N = 307)	182 (59.3)	125 (40.7)	
Other species (N = 2)	1 (50)	1 (50)	
Species details not available (N = 38)	24 (63.2)	14 (36.8)	
*Group of ethnicities*			< 0.001
Highland Papuans (N = 99)	47 (47.5)	52 (52.5)	
Lowland Papuans (N = 147)	76 (51.7)	71 (48.3)	
Non-Papuans (N = 361)	263 (72.9)	98 (27.1)	
*Time between trial enrolment and survey*			0.417
Before/at 6-month follow-up (N = 195)	129 (66.2)	66 (33.9)	
After 6-month follow-up (N = 412)	257 (62.4)	155 (37.6)	

*Measure of adherence was calculated from survey participants who said they finished all or some of the drugs given during trial/in the last malaria episode AND mentioned both ‘blue drugs’ PLUS any of the following: ‘brown drugs,’ ‘small drugs,’ ‘spleen drugs,’ or ‘primaquine’

**p-values were calculated with Fisher’s exact test for malaria species and chi-square tests for the other variables

In both the supervised and unsupervised arms *all* patients were given 14-day PQ regardless of malaria species whereas in the control arm only those with single or mixed *P. vivax* or *P. ovale* infections received 14-day PQ. For this reason, whether adherence differed with the species of malaria at enrolment was explored. There was no significant difference between malaria diagnosis and self-reported adherence (p = 0.084, Table 6). There was a significant difference between the different ethnicities in terms of reported adherence (p < 0.001), with both highland (Amungme, Damal, Dani, Mee, Moni, Nduga, Wamena) and lowland (Kamoro, Asmat, Biak, Fakfak, Jayapura, Merauke, Moor, Serui, Sorong) Papuans reporting lower adherence: 47.5% (47/99) and 51.7% (76/147) for highland and lowland Papuans, respectively than non-Papuans (72.9%; 263/361, Table 6).

Malaria species at enrolment (Fisher’s exact test p = 0.011) and ethnicity (*chi-*square test p = 0.005) differed significantly between the study arms. In the post hoc analysis using the Marascuilo procedure, the difference in adherence between unsupervised and control groups and between highland-Papuan and non-Papuan ethnic groups, remained significant.

#### Reported influence of supervision strategy on adherence

In the supervised arm, qualitative inquiries indicated that both trial participants and field supervision staff considered that supervised treatment on alternate days was suboptimal because participants were dependent on meeting field supervision staff to receive their tablets. If they were not reached at home for supervision visits, they could not take the PQ dose for that day. The qualitative strand also revealed that language barriers complicated the social relations between participants and field researchers of different ethnicities, since some Papuans spoke little Indonesian and few field researchers spoke any of the local languages. Moreover, high population mobility among certain ethnicities, such as travelling for mangrove forest fishing (Kamoro) and travelling between mountain villages and the mine (Dani, Damal, Moni), complicated both the supervision of the adherence by field researchers and the adherence by participants.

## Discussion

This mixed-methods study investigated adherence to the radical cure treatment of *P. vivax*, including both a 3-day regimen of DP plus a 14-day regimen of PQ in Papua’s Mimika District, Indonesia. The findings highlight that in this location, (i) ethnicity is a strong predictor of adherence and influenced the effectiveness of treatment supervision; (ii) perceptions of and experiences with different healthcare providers, together with (iii) perceptions of medicines, influenced the *process* of treatment adherence.

(i) *Ethnicity* In Papua, the perceptions of medicines and of malaria must be interpreted in the context of a remarkably diverse ethnic setting. Furthermore, the presence of a local mine dominates the socioeconomic landscape. As is frequently found in mining communities, the mine attracts people from other parts of the country to form the main mine workforce [31], connecting local and non-local workforces with the same healthcare services. There were interethnic differences in adherence to malaria medication, with lower adherence among Papuan populations as compared to non-Papuan ethnicities. The qualitative strand of this study shows that ethnicity-related structural factors could explain the interethnic difference in treatment adherence. First, language barriers complicated an efficient dialogue between healthcare workers and patients enabling a shared understanding of the importance of adherence to *P. vivax* malaria treatment [24]. Many healthcare workers are not Papuan and do not speak any of the local languages. Second, Papuan populations are socially and economically marginalized, with lower educational and employment opportunities compared to non-Papuans [26]. Issues of trust and hierarchical healthcare worker-patient relationship—that are well documented in other health interventions—can contribute to explaining lower adherence in highland and lowland Papuans [9]. Third, the mobility associated with mining work and the subsistence strategies practiced by both highland and lowland Papuans are influencing the feasibility of adhering to long duration treatments. Treatment supervision and encouragement provided by someone living in the same village or neighborhood (for example a community health worker)—adapting both the visiting hours and the explanation about the medicines to the patient’s socio-ethnic context—has potential to minimize this problem.
(ii) *Perceptions of and experiences with different healthcare providers* In this complex social context, people often self-diagnose malaria based on symptoms and usually only visit a health provider more than 24 h after onset of symptoms, with the purpose of confirming their self-diagnosis and to receive treatment. The high prevalence of malaria often makes people consider malaria when they have a fever, and this prompts the expectation of a positive diagnosis and associated DP treatment when they visit a health facility. The aversion to having a negative diagnosis and hence not receiving treatment is compounded by the fact that examination for other probable causes of fever is rarely done at any clinic. Gaps in diagnosis for acute febrile illness at primary healthcare settings are common in the global south [32–34]. In Indonesia, like in many other countries, few studies have investigated the epidemiology of febrile illness etiologies [35]. Availability of point-of-care diagnostic tools for other probable causes of fever would lead to more efficient malaria control [36].
  In this setting, the mining company-funded health providers represent an important alternative to public health providers, as they have existed for longer and were perceived as more likely to provide a positive diagnosis than public health centers. The choice of health provider is also driven by factors of familiarity, cost of treatment and transport, waiting time, and perceived quality of service [37, 38]. Prior to a visit to a health provider, the severity of the symptoms was usually assessed by a self-treatment with drugs leftover from previous illnesses or with over-the counter drugs [39, 40]. Herbal topical and oral remedies were complementary medicines used to alleviate symptoms rather than replace biomedical treatment [41].
(iii) *Perception of medicines* Local healthcare workers and community members differentiated between two types of malaria (*tropika* and *tersiana*), against which ‘blue drugs’ (referring to DP) were perceived to be efficacious while the ‘brown drugs’ (referring to PQ) were considered less potent, associated to vitamins or supplements. PQ had been around long before DP was introduced as a malaria treatment and has accompanied different drugs that were used as treatment for acute stage malaria. However, DP was invariably perceived as malaria drug and the self-reported adherence to DP was far better than to PQ. This suggested a high level of acceptance of DP as a new drug introduced in 2006 to replace other schizontocidal drugs that were inefficacious due to resistance [42, 43].

Inconsistent prescribing of PQ by healthcare providers and the long duration of PQ treatment helped shape conceptualizations of PQ as supplements rather than as malaria treatment. Since 2006, clinical guidelines have recommended that patients diagnosed with *P. falciparum* get a single dose of PQ, whilst patients diagnosed with *P. vivax* malaria get a 14-day low dose PQ regimen. Inconsistent and inadequate dosage of PQ prescriptions also happened due to the unfamiliarity of health workers with treatment guidelines or due to supply shortages [30]. In the context of this trial, the recommended low dose was used in the control arm, while the supervised and unsupervised arms were treated with a higher dose PQ regimen. Without supervision, the challenges of the long duration and higher number of daily tablets received by participants in the unsupervised arm could consequently have reduced adherence.

This study also found that the physical appearance of the PQ (smaller size and earthy colour) in contrast to DP evoked a perception of it being less important. The relationship between drug colour and size and perceived efficacy has been studied in other settings, and the colour and size that is perceived to be “strong” might vary across cultures [44, 45]. An appearance that evoked a perception of a drug being “strong” could prove essential in increasing drug adherence.

### Limitations

This study has several limitations. The sample only included patients recruited from public health centres into a clinical trial and this may have introduced selection and participation bias. Although very few patients screened for enrolment were subsequently excluded from the study, caution is needed in over-interpreting the study results particularly regarding treatment seeking behaviour. The proportion of community members that prefer mining company-funded health providers might be higher and there may be a part of the community that was not sampled since they did not access healthcare despite having malaria. Most of the study population were young (as expected as malaria is more prevalent in children), however the treatment-seeking behaviour for adult or child patients (through their guardian) were not differentiated. This study did not conduct multivariable analysis to quantify potential interactions between the factors influencing adherence. Other potential confounders such as gender and economic status were also not analysed in this paper.

## Conclusion

Although oral supervision of a 14-day PQ course for *P. vivax* malaria may improve adherence to a prolonged treatment regimen, the supervision strategy was challenged by both socio-cultural factors (language and other interethnic encounter barriers, high mobility of patients of certain ethnicities/occupations, perceptions of medicines) and practical bottlenecks (dosage change, difficult implementation of home visits by fieldworkers to remote locations) resulting in lower adherence among indigenous populations compared to non-indigenous populations. Both socio-cultural logics and practical challenges that underlie patient decisions to adhere to or to abandon PQ treatment are important to address. The lack of representation of indigenous populations among healthcare workers is a likely contributor. Structural barriers that hinder the process of patient adherence are crucial to consider in the development and roll-out of effective malaria treatment policies such as oral supervision strategies.

## Supplementary Information

**Additional file 1. Table S1. **Demographic characteristics of survey participants

## Data Availability

All data generated or analyzed in the quantitative strand of this study are included in this published article [and its supplementary information files]. The data that support the findings and analyzed in the qualitative strand of this study are available from the corresponding author on reasonable request.
