# Supplementary material for: Adherence to 14-day radical cure for Plasmodium vivax malaria in Papua, Indonesia: a mixed-methods study
Source: Malar J. 2023 May 20;22:162. doi: 10.1186/s12936-023-04578-3 (PMC10199529; doi:10.1186/s12936-023-04578-3)
Supplement: Supplementary file 1 — Additional file 1. Table S1. Demographic characteristics of survey participants [file 12936_2023_4578_MOESM1_ESM.docx]

**Additional Materials**

**Table S1. Demographic characteristics of survey participants**

| **Variables** | **Frequency** | **Percent** |
| --- | --- | --- |
| **Demographic characteristic (N = 607)** |  |  |
| ***Female sex*** | 306 | 50.4 |
| ***Age***, median (IQR) | 17 (8-33) |  |
| **Religion** |  |  |
| Catholic | 193 | 31.8 |
| Christian (various denominations) | 218 | 35.9 |
| Muslim | 196 | 32.3 |
| **Main occupation** |  |  |
| Student | 230 | 37.9 |
| Home maker/stay-at-home spouse | 93 | 15.3 |
| Farming | 88 | 14.5 |
| Has not started school (young children) | 83 | 13.7 |
| Unspecified private employee | 28 | 4.6 |
| Driver | 13 | 2.1 |
| Civil servant and temp. government employee | 13 | 2.1 |
| Sales (shop, food vendors, etc.) | 13 | 2.1 |
| Day laborer | 11 | 1.8 |
| Mining and mining company work | 10 | 1.6 |
| Fishing | 4 | 0.7 |
| **Location of interview** |  |  |
| Home | 506 | 83.4 |
| Elsewhere (school, workplace, market, etc.) | 101 | 16.6 |
| **Housing condition (N = 506)** |  |  |
| **Residence ownership** |  |  |
| Their own home | 351 | 69.4 |
| Family house | 128 | 25.3 |
| Renting | 73 | 14.4 |
| Work accommodation | 28 | 5.5 |
| Church accommodation | 5 | 1.0 |
| **Housing structure** |  |  |
| House on stilts | 123 | 24.3 |
| ***Floor*** |  |  |
| Cement | 199 | 39.3 |
| Ceramic tile | 187 | 37.0 |
| Wood planks | 111 | 21.9 |
| Dirt/earth | 9 | 1.8 |
| ***Wall*** |  |  |
| Concrete | 298 | 58.9 |
| Wood | 205 | 40.5 |
| Half concrete-half wood/stone/thatch | 3 | 0.6 |
| ***Roof*** |  |  |
| Tin | 503 | 99.4 |
| Thatch | 3 | 0.6 |
| **Animals raised and kept** (pigs/cows/goats/chicken/etc.) | 328 | 64.8 |
| ***Pig count*** |  |  |
| No pigs | 482 | 95.3 |
| One pig | 30 | 5.9 |
| 2-5 pigs | 66 | 13.0 |
| 6-20 pigs | 29 | 5.7 |
